# Evaluation of serum C4d levels in patients with systemic lupus erythematosus and its relation to lupus nephritis

**DOI:** 10.22088/cjim.14.2.231

**Published:** 2023

**Authors:** Nayereh Saadati, Maryam Miri

**Affiliations:** 1 Department of Internal Medicine, Mashhad University of Medical Sciences, Mashhad, Iran; 2 Kidney Transplantation Complications Research Center, Mashhad University of Medical Sciences, Mashhad, Iran

**Keywords:** Lupus nephritis, Systemic lupus erythematosus, C4d

## Abstract

**Background:**

Lupus nephritis (LN) is a debilitating complication of systemic lupus erythematosus (SLE). Renal biopsy is the gold standard for evaluating LN. Serum C4d is a potential non-invasive method for evaluating LN. The purpose of this study was to evaluate the value of C4d in the assessment of LN.

**Methods:**

This cross-sectional study was conducted on patients with LN who were referred to a tertiary hospital in Mashhad, Iran. Subjects were divided into four groups including LN, SLE without renal involvement, chronic kidney disease (CKD) and healthy controls. Serum C4d. creatinine, and glomerular filtration rate (GFR) were assessed for all subjects.

**Results:**

Forty-three subjects (11, 25.6% healthy controls, 9, 20.9% SLE patients, 13, 30.2% LN and 10, 23.3% CKD patients) participated in this study. CKD group were significantly older than other groups (p>0.05). There was a significant difference in gender distribution between groups (p<0.001). Median serum C4d were 0.6 in healthy controls and CKD group and 0.3 in SLE and LN groups. There was no significant difference in serum C4d between groups (p=0.503).

**Conclusion:**

The findings of this study indicated that serum C4d might not be a promising marker in the assessment of LN. These findings should be documented by further multicenter studies.

Systemic lupus erythematosus (SLE) is an autoimmune disease that causes inflammatory reactions in all body organs (1). Kidney is a common site for these immune reactions during SLE (2). Lupus nephritis (LN) occurs in nearly 50% of SLE patients (2). The prevalence of LN was reported to be higher among the Black Americans compared to the White Americans (3). Furthermore, LN was reported to occur at lower age and had more complications among Black Americans compared to White Americans (3). This finding might indicate a genetic susceptibility to LN among some races (4).It was reported that 48% of the Iranian SLE patients develop LN and that half of the LN patients were in progressive stages of glomerulonephritis (5). The response to treatment is poor in progressive stages of LN, therefore, it is necessary to diagnose LN at early stages in order to prevent renal failure and end-stage renal disease (ESRD). Renal involvement can be assessed by urinalysis but this method is not enough for the management of LN (6). Renal biopsy has an important role both in defining type of renal involvement and in ruling out differential diagnoses (6). Although renal function might improve by immunosuppressive treatment in SLE, but studies have shown that renal impairment still existed in renal biopsies (2, 7).

Therefore, it is essential to perform serial renal biopsies to assess response to treatment, extent of renal involvement and thus decide on the management of LN. Although renal biopsy is a gold standard in diagnosis of many kidney injuries, it is invasive and might accompany with complications (8, 9). Some occasionally uncommon complications or renal biopsy are concerning. These complications include bleeding, infection, and peritonitis due to intestinal rupture, acute kidney stenosis, septicemia and death (8-11). Complement system activation is a distinct characteristic of SLE. Therefore, SLE activity has long been assessed based on the evaluation of serum levels of C3 and C4 (12, 13). Serum C3 and C4 levels depend on synthesis and activation of these components. Therefore, measuring the products of the activation of C3 and C4 can also act as a marker for SLE activity (14).

 It was recently found that serum levels of C3 and C4 have low sensitivity in the follow up of SLE patients (14). A newly proposed marker for the assessment of SLE activity is C4d. C4d is a stable protein product from C4. C4d is produced during the activation of C4 through classic and lectin pathways (15). It was reported that C4d is a reliable marker for LN and has higher precision compared to C3 and C4 (16). Previous studies assessed the relationship between C4d on erythrocyte or platelets and found that C4d on erythrocytes was significantly related to the severity of SLE (17, 18). In another study C4d deposition in renal tissue was found to be associated with LN (19). These methods are expensive and require expertise and special equipment. On the other hand, serum c4d assessment is cheaper and can be performed widely in hospitals.

 Therefore, serum C4d assessment can be considered as a potential marker for the assessment of LN if its reliability is documented. Clinical evidence regarding the value of C4d in the assessment of LN is scarce. Therefore, the aim of this study was to assess the relationship between serum C4d level and the determinants of renal function in LN.

## Methods

This study was a cross-sectional study that was conducted on three case groups, including SLE patients with or without chronic kidney disease (CKD), chronic kidney disease due to other causes than SLE, and a healthy control group from March 2018 to March 2019.The study protocol was approved by the ethics Committee of the Mashhad University of Medical Sciences (Code: IR.MUMS.MEDICAL.REC.1397.515). All subjects gave a written informed consent before participating in the study.

Sample size was calculated based on the findings of the study by Martin et al. (2017) based on two sample t-test analysis by considering 5% type I error and 10% type II error (16). The calculated sample size was 15 patients in each group. Three groups of patients who were referred to the Quaem Hospital, Mashhad, Iran were selected along with a healthy control group.

The case groups included patients with documented diagnosis of SLE without renal involvement, patients with documented diagnosis of CKD due to SLE, and patients with documented diagnosis of CKD due to other causes. Healthy controls, who did not have a documented diagnosis of SLE or CKD due to any cause, were selected among. Subjects were excluded if they had rheumatologic comorbidities and renal involvement due to other glomerular diseases. Venous blood samples were obtained from all subjects and transferred to laboratory. Samples were stored at -70 ᵒC till the time of evaluation. C4d level was assessed using the c4d Elisa kit. SLE activity was assessed based on Systemic Lupus Erythematosus Disease Activity Index. (SLEDAI) scoring system. LN was defined as renal involvement in SLE patients based on urine sedimentation or increased serum creatinine (Cr); or based on LN diagnosis in renal biopsy. 

***Statistical analysis: *** The statistical package for social sciences (SPSS) software version 23 was used to analyze data. Continuous variables were compared between groups using One-way analysis of variance (ANOVA) with Brown-Forsythe test for the equality of variance and Games Howell as post hoc test for parametric variables and Kruskal-Wallis test with Mann-Whitney test for pairwise comparison for non-parametric variables. Categorical variables were compared between groups using the Monte Carlo test. In order to assess the ability of C4d in predicting lupus nephritis, the ROC analysis was performed on age adjusted C4d levels. Level of statistical significance was defined as p<0.05. Data were analyzed by SPSS 25 using chi-square, Kruskal-Wallis and Mann-Whitney statistical tests. A p-value <0.05 was considered statistically significant.

## Results

A total of 43 patients, including 11 (25.6%) healthy controls, 9 (20.9%) SLE patients without nephritis, 13 (30.2%) LN and 10 (23.3%) CKD patients, participated in this study. There was a significant difference between groups in terms of gender distribution (p<0.001) (Table 1).The male to female ratio (M: F) was 4.5:1 in the healthy control group, while the M: F was 0:9 in the SLE group, 0.18:1 in the LN group and 0.43:1 in the CKD group. After categorizing patients based on serum Cr into Cr<1 mg/dl and Cr>1 mg/dl, there was a significant difference between groups (p=0.023) (Table 3). 

**Figure 1:**
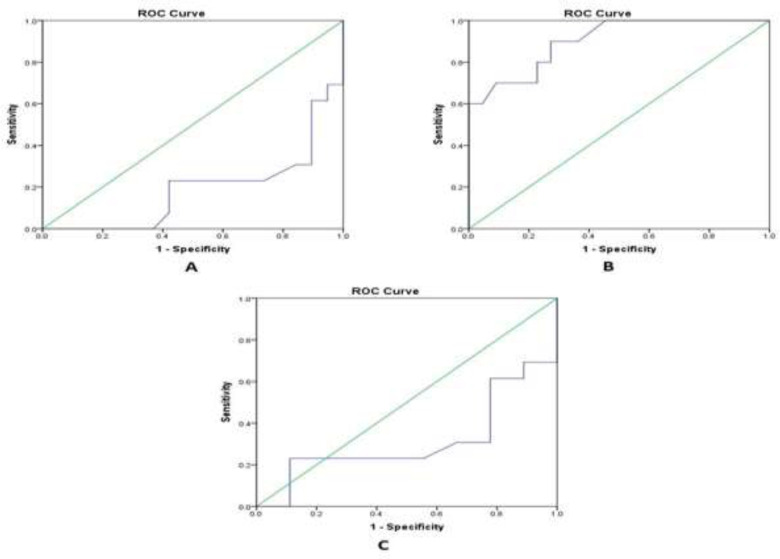
The ROC for C4d in detecting LN (A) and CKD (B) among SLE patients. ROC for C4d in detecting LN among SLE patients excluding those with CKD (C)

This indicates that the serum Cr distribution pattern significantly differed between CKD and SLE (p=0.001) and LN (p=0.007) groups (Table 2). The ROC analysis was performed on SLE patients without LN, SLE patients with LN and SLE patients with CKD (Figure 1 A and B). The area under curve for detecting LN was 0.812, p=0.003 and for detecting CKD was 0.902, p<0.001. The C4d cut-off for detecting LN was 1.7086 with the sensitivity and specificity of 76.9% and 73.7%, respectively. The C4d cut-off for detection of CKD was 1.7128 with the sensitivity and specificity of 90.0% and 72.7%, respectively. The ROC analysis was used to assess whether C4d can differentiate LN among SLE patients excluding the CKD patients (Figure 1 C). The area under curve was 0.688, p=0.142. The C4d cut-off for detection of LN was 1.7246 with the sensitivity and specificity of 76.9% and 88.9%, respectively. 

**Table 1:** Comparison of gender distribution in the study patients

**Gender**	**Total** **Frequency (%)**	**Normal** **n=11**	**Lupus** **n=9**	**Lupus nephritis** **n=13**	**Chronic Kidney Disease** **n=10**	**p**
**Male, N (%) **	14 (32.6)	9 (81.8)^abc^	0 (0.0)^a^	2 (15.4)^b^	3 (30.0)^c^	<0.001**
**Female**	29 (67.4)	2 (18.2)	9 (100.0)	11 (84.6)	7 (70.0)	

The Monte Carlo test was used for the assessment of the relationship between gender and diagnosis due to the presence of more than 20% cells with values less than 5

^a^ p<0.001, ^b^ p=0.003

** Significant association

**Table 2:** Comparison of study variables between groups

**Variable**	**Total** **N=43**	**Normal** **n=11**	**Lupus** **n=9**	**Lupus nephritis** **n=13**	**Chronic Kidney Disease** **n=10**	**p**
**GFR (ml/min)**	69.29 (58.67)†	83.76±7.68^ab^	80.91±21.73^bc^	55.23±33.63^ad^	20.07±8.69^acd^	<0.001**‡
**Cr (mg/dl)**	1.03 (1.50)†	0.99±0.12^e^	0.70 (0.95)^f^	1.10±1.04†^eg^	2.65 (10.50)†^efg^	<0.001**Ɨ
**Age (years)**	36.00 (18.00)†	38.27±6.69^hk^	39.00±9.05^i^	32.00 (19.00)†^jk^	61.20±16.71^ hi j^	<0.001**Ɨ
**C4d (mg/l)**	0.60 (0.60)†	0.60 (0.10)†	0.30 (1.80)†	0.30 (1.40)†	0.60 (0.50)†	0.503

Cr: creatinine, mg: milligram, ml: milliliter, dl: deciliter, l: liter, min: minute, GFR: glomerular filtration rate

† Median and interquartile range were presented

‡ One-way analysis of variance with Brown-Forsythe test for the equality of variance with Games Howell as post hoc test.

Ɨ The Kruskal-Wallis test was used for the comparison and the Mann-Whitney test was used for pairwise comparison

^a^ p=0.046, ^b^ p<0.001, ^c^ p<0.001, ^d ^p<0.001, ^e^ p<0.001, ^f^ p=0.001, ^g ^p=0.001, ^h^ p=0.004, ^I^ p<0.001, ^j^ p<0.001, ^k^ p=0.021

** Significant difference

## Discussion

The role of C4d in the assessment of LN is a new subject in the assessment of SLE. This method is less invasive than renal biopsy in the assessment of LN. There is scarcity of data regarding the relationship between serum C4d levels and LN parameters. Therefore, this study was conducted to assess serum C4d levels in patients with SLE without renal involvement, LN patients and to compare serum C4d between these two groups as well as patients with CKD due to other causes and healthy controls.

The findings of this study revealed no significant difference was observed in terms of serum C4d between groups. The current study observed the highest median C4d levels in patients with CKD and that no significant difference was observed between SLE patients without renal involvement and LN patients. This finding was in line with the findings of a previous study, that showed although serum c4d levels were not significantly different in LN patients compared to SLE patients without renal involvement although they reported that serum c4d level was higher in patients with active LN compared to patients in the remission phase (20).The current study only included patients with active LN, therefore, comparison of serum c4d levels between patients with active LN and those in the remission phase could not be performed. In contrast to the findings of the current study, serum levels of c4d (5.19 mg/l) were higher in the mentioned study compared to the current study (0.3 mg/l) (20). This difference in the serum C4d levels might be reason for the observed lack of statistical significance in comparing serum C4d levels between groups in the current study. Furthermore, the study reported that the serum level of C4d was significantly higher in LN patients compared to healthy controls (20). Similarly, in a study on 69 SLE patients, serum c4d levels were found to be significantly higher in SLE patients compared to healthy controls (16). The mean serum C4d was 0.49 mg/l in the study which was slightly higher than the observed median serum c4d levels in the current study. Unlike the mentioned studies, the current study revealed that serum C4d levels were higher in healthy controls compared to both patients with SLE without renal involvement and patients with LN. The reason for this difference might be due to the difference in the C4d kit used in the current study, as evidenced by a lower reading for all groups compared to the mentioned studies. Furthermore, the sample size in the current study was lower than both the studies mentioned before. This difference in sample size resulted in reduced power of the statistical analysis in the current study. On the hand, the distribution of the conditions in the study groups was not proportionate, which might have affected the findings of the current study. In a study on 72 Iranian SLE patients in 2018, serum C4d levels were found to have no significant relationship with SLE activity and LN assessment parameters (21). This finding was in line with the findings of the current study and may indicate a genetic difference in serum C4d levels among SLE patients. Although this hypothesis should be tested in multicenter studies with larger sample sizes.

In contrast to the findings of the current study, the study by Kraaij et al. (2019) on 50 SLE patients (22). They reported that serum C4d level was significantly related to proteinuria in SLE patients (22). A reason for the difference between the study by Kraaij et al. (2019) and the current study might be due to the difference in the reported median serum c4d levels. The median serum C4d level was 1.25 mg/l in the study by mentioned study which was higher than the observed 0.3 mg/l and 0.6 mg/l in LN and CKD patients, respectively, in the current study. Furthermore, the sample size in the study by Kraaij et al. (2019) was larger than the sample size in the current study. The current study showed that C4d can be used in determining CKD and LN from other kidney involvements in SLE, while it could not effectively differentiate LN in SLE patients after excluding CKD.

 Considering the small sample size of the current study, the area under curve was used as an indicator of effect size (23). Considering the report that equated AUC of 64% to Cohen’s d of 0.50 (23), only the ROC findings in terms of CKD differentiation had highly acceptable effects size based on area under curve and the findings of the current study in this regard might not change if the study was performed on a larger sample size. Therefore, the findings of this study may not have the required power to reject the findings of previous studies in terms of a relationship between C4d levels and LN. One of the strengths of the current study was the inclusion of CKD and healthy individuals as controls along with SLE patients in the assessment of serum C4d levels. This design provided a better picture regarding the distribution of serum c4d in different patient populations. On the other hand due to the small number of patients in the study groups, the findings of the current study might not be generalizable to SLE patients with LN. Therefore, it is recommended that studies with larger sample sizes should be conducted to assess the reliability and validity of serum C4d levels in the assessment of LN.
